# Challenges in Implementing Community-Based Healthcare Teams in a Low-Income Country Context: Lessons From Ethiopia’s Family Health Teams

**DOI:** 10.34172/ijhpm.2021.52

**Published:** 2021-06-07

**Authors:** Teralynn Ludwick, Misganu Endriyas, Alison Morgan, Sumit Kane, Margaret Kelaher, Barbara McPake

**Affiliations:** ^1^Nossal Institute for Global Health, Melbourne School of Population and Global Health, University of Melbourne, Melbourne, VIC, Australia.; ^2^Health Research and Technology Transfer Office, SNNPR Regional Health Bureau, Hawassa, Ethiopia.; ^3^Centre for Health Policy, School of Population and Global Health, University of Melbourne, Melbourne, VIC, Australia.

**Keywords:** Healthcare Teams, Health Human Resources, Urban, Community Health Workers, Non-communicable Diseases (NCDs), Ethiopia

## Abstract

**Background:** Addressing chronic diseases and intra-urban health disparities in low- and middle-income countries (LMICs) requires new health service models. Team-based healthcare models can improve management of chronic diseases/complex conditions. There is interest in integrating community health workers (CHWs) into these teams, given their effectiveness in reaching underserved populations. However healthcare team models are difficult to effectively implement, and there is little experience with team-based models in LMICs and with CHW-integrated models more generally. Our study aims to understand the determinants related to the poor adoption of Ethiopia’s family health teams (FHTs); and, raise considerations for initiating CHW-integrated healthcare team models in LMIC cities.

**Methods:** Using the Consolidated Framework for Implementation Research (CFIR), we examine organizational-level factors related to implementation climate and readiness (work processes/incentives/resources/leadership) and system-level factors (policy guidelines/governance/financing) that affected adoption of FHTs in two Ethiopian cities. Using semi-structured interviews/focus groups, we sought implementation perspectives from 33 FHT members and 18 administrators. We used framework analysis to deductively code data to CFIR domains.

**Results:** Factors associated with implementation climate and readiness negatively impacted FHT adoption. Failure to tap into financial, political, and performance motivations of key stakeholders/FHT members contributed to low willingness to participate, while resource constraints restricted capacity to implement. Workload issues combined with no financial incentives/perceived benefit contributed to poor adoption among clinical professionals. Meanwhile, staffing constraints and unavailability of medicines/supplies/transport contributed to poor implementation readiness, further decreasing willingness among clinical professionals/managers to prioritize non-clinic based activities. The federally-driven program failed to provide budgetary incentives or tap into political motivations of municipal/health centre administrators.

**Conclusion:** Lessons from Ethiopia’s challenges in implementing its FHT program suggest that LMICs interested in adopting CHW-integrated healthcare team models should closely consider health system readiness (budgets, staffing, equipment/medicines) as well as incentivization strategies (financial, professional, political) to drive organizational change.

## Background

**Key Messages**

**Implications for policy makers**

- Community-based healthcare teams offer potential to better manage chronic diseases and respond to the multifaceted health needs of vulnerable populations, but are challenging to implement and need appropriate resourcing.
- Our study shows that low- and middle-income country (LMIC) community-based healthcare team models face important barriers to adoption, particularly around motivation and incentives, which are driven strongly by resource constraints. Clinical professionals and managers showed low commitment to participating in and supporting teams. As such, LMICs need to carefully consider resource requirements and institute organizational change management strategies to support successful adoption of team models.
- For many LMICs, it may be more practical to first implement healthcare team models, especially those involving community health workers (CHWs), in urban settings where there are higher numbers of healthcare workers and better-equipped facilities to support teams.

**Implications for the public**

Creating teams of healthcare professionals has the potential to improve treatment and support for people with chronic diseases and other complex conditions. However, establishing healthcare teams can be challenging, and there is little guidance for low- and middle-income countries (LMICs) on how to create an enabling environment to support their introduction. By outlining challenges encountered during early implementation of a team-based model in two Ethiopian cities, our study provides important lessons that can inform more successful introduction of teams in LMICs in the future. Despite strong evidence that CHWs help reach and connect under-served populations with healthcare, there were difficulties integrating CHWs into Ethiopia’s family health teams (FHTs). Findings from our study demonstrate the importance of resourcing, management of teams, and attitudinal change among providers in order to support team models in an LMIC context.

Growing links between urbanization, inequality, and non-communicable disease risks are giving rise to new patterns of health disadvantage among the urban poor in low- and middle-income countries (LMICs).^1,2^ However, primary healthcare systems in many LMICs are not well-oriented to reach the urban poor or manage chronic diseases effectively.^3,4^ New practice models are required to respond to these challenges.^5,6^

 Team-based healthcare offers potential to better manage chronic diseases and complex conditions.^7,8^ Team-based healthcare is “the provision of health services to individuals, families, and/or their communities by at least two health providers who work collaboratively with patients and their caregivers—to the extent preferred by each patient—to accomplish shared goals within and across settings to achieve coordinated, high-quality care.”^9^ With many health organizations now endorsing community-based, healthcare team models,^10,11^ there is interest in integrating community health workers (CHWs) into these teams, given their effectiveness in reducing health disparities among vulnerable and underserved populations in high and low-income countries alike.^12^ However, the integration of CHWs in healthcare teams is not well studied in any setting,^8^ and experience with healthcare teams in LMICs is limited. Ethiopia’s family health teams (FHTs) represent one of the first efforts by a low-income country to initiate a community-based healthcare team model; the FHTs include CHWs and focus on providing services to manage chronic diseases and other conditions among the poor and other vulnerable groups in Ethiopian cities.

 While collaboration in healthcare offers many advantages, including improved quality of patient care, resource efficiency, effective workload management, and enhanced staff satisfaction and retention,^13-15^ implementation of teamwork models in healthcare constitutes a significant organizational change, often resulting in a contested and difficult adoption process.^16,17^ Organizational change models suggest that factors associated with organizational structure and culture (eg, professional hierarchies), management and leadership, and the external environment (eg, policy and funding context) influence the adoption of innovations.^18-21^ The adoption phase is a critical first step in organizational change theories^19^ and in the healthcare team literature.^17^ Reviews of facilitators and barriers of teamwork in healthcare highlight the need to examine factors that influence ‘enrolment’ – known as ‘adoption’ in the implementation research literature – in order to understand what affects initial willingness to participate.^17^ However, very few implementation studies have examined this critical stage.^22^ Existing studies have tended to focus on interpersonal dynamics with few comprehensively investigating the broader implementation environment, particularly organizational and system determinants, that may affect adoption.^23^

 With limited evidence to guide LMICs, our study critically examines the poor adoption and rapid fade-out of Ethiopia’s FHTs in two cities. Our objectives are to: (1) comprehensively identify determinants of (non)adoption of the FHTs; and, (2) outline considerations for initiating community-based team healthcare models in LMICs. The findings provide valuable insights into the factors that shape adoption and early implementation of team-based healthcare delivery models in LMIC contexts, with specific lessons for teams involving CHWs.

###  Setting

 Ethiopia is among the fastest urbanizing countries, with the urban population expected to triple from 2012-2037 to reach 42 million.^24^ Chronic diseases now account for 39% of all deaths in Ethiopia, present significant economic, health, and social costs, and represent a growing policy priority.^25,26^

 In 2014/2015, the federal government launched a primary healthcare reform to strengthen community-based, urban service provision for low-income households, including those with chronic diseases.^27^ These reforms included the launch of FHTs which integrate clinical professionals into outreach teams with CHWs, known locally as urban health extension professionals (UHEPs). UHEPs are female, diploma-level nurses (10th grade education plus 3 years of college) who provide door-to-door health education and referrals within their catchment of approximately 500 households.

 The reform called for 3-5 FHTs to be established per health centre in selected pilot cities and to include: a family health doctor/health officer/Bachelor-degree-holding nurse as lead; 5-6 UHEPs; a social worker; and, laboratory, pharmacy and administrative staff as needed. On alternating days, FHTs were to be assigned either to outreach visits or to receiving FHT-referred clients in a dedicated outpatient room.^27^ FHTs focus on prioritized households categorized by income and health needs (pregnant women/young children; those with chronic diseases; elderly/bedridden). Using standardized forms, UHEPs carried out surveys with all catchment households to document health conditions and identify low-income households. Using this information and their own discretion, UHEPs selected households most in need of FHT visits. Within our primary study site, the number of households per FHT catchment (neighbourhood) ranged from around 700 to 2500, and UHEPs identified from 200 to 2200 households as low-income and as having one of the three priority health conditions.

 As part of FHT formation, the Ministry of Health recommended that UHEP’s duty station be moved from *Kebele *administrative offices (the lowest level of municipal administration) to health centres. This physical move also entailed health centre-based administrators taking on primary responsibility for supervising UHEPs and UHEPs reporting directly to them. FHT members, health centre administrators, and city administrators attended a two-day training that addressed the program aims and provided practice visits. Teams were to be supplied with blood pressure monitors, glucometers, first aid kits and medicines as needed.

 The FHTs’ are intended to: (1) improve health outcomes (by providing free, doorstep treatment for low-income households and other vulnerable populations); (2) strengthen community-to-facility referrals; and (3) respond to performance challenges faced by UHEPs, including weak health centre linkages, lack of professional development, low motivation, and difficulties serving all catchment households.^28,29^ Figure presents the program logic.

**Figure:**
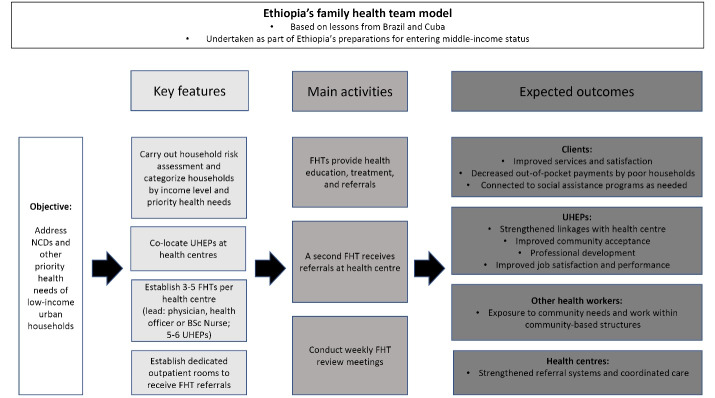
Key Features, Activities and Expected Outcomes of Ethiopia’s FHTs. Abbreviations: NCDs, non-communicable diseases; FHT, family health team; UHEPs, urban health extension professionals; BSC, Bachelor of Science.

 The FHTs were originally piloted in the capital city, Addis Ababa and then extended to 5 regional cities. In this second wave of implementation, one health centre in each of the 5 cities was selected by the respective Regional Health Bureau. Implementation was further extended to additional cities in subsequent waves.

## Methods

 As one of the pioneering, low-income countries to trial a team-based approach in healthcare, our study examines the early phases of FHT implementation in Ethiopia. Given the rapid fade-out of the FHTs (within a few short months), we focus specifically on the determinants of adoption – defined as the willingness to implement a program.^30,31^ As one of the pioneering, low-income countries to trial a team-based approach in healthcare, our study examines the early phases of FHT implementation in Ethiopia. Given the rapid fade-out of the FHTs (within a few short months), we focus specifically on the determinants of adoption – defined as the willingness to implement a program.^30,31^ We use a qualitative, implementation research approach to examine participants’ perspectives on FHT adoption. In our study, we examine:

1. What setting-level determinants affected the (non)adoption of FHTs at health centres? Are these determinants primarily related to the organizational setting and external environment?
2. What are key health system considerations for initiating community-based healthcare team models in LMICs?

###  Conceptual Framework

 Given limited experience with healthcare team models in LMICs, we used the Consolidated Framework for Implementation Research (CFIR) to guide our approach.^32^ As a ‘meta-theoretical’ framework, the CFIR provides a ‘menu of constructs’ related to both the inner and outer setting that are associated with effective implementation without predefining specific hypotheses or interrelationships between different determinants and ecological levels.^32^ The broad-based nature of the CFIR and lack of pre-identified relationships suited our aim to explore how an LMIC-context influences the implementation of community-based, healthcare teams. The CFIR comprises five domains that may affect an intervention’s implementation: intervention characteristics, inner setting (organizational-level factors including implementation climate and readiness), outer setting (broader influencing factors, including resourcing and external mandates), characteristics of individuals, and implementation process (Table 1).

**Table 1:** CFIR Domains

**Characteristics of individuals: **Characteristics of individuals involved in implementation that might influence implementation. Five included constructs are: knowledge and beliefs about the intervention, self-efficacy, individual stage of change, individual identification with the organization, other personal attributes.
**Intervention characteristics: **Aspects of an intervention that may impact implementation success, including evidence quality and strength, relative advantage, adaptability, trialability, complexity, design quality and presentation, and cost.
**Inner setting: **Features of the implementing organization that might influence implementation. Twelve constructs are included in inner setting: compatibility and relative priority of the intervention, team culture, structures for goal-setting and feedback, leadership engagement, and the implementation climate. Implementation climate and implementation readiness contain multiple sub-domains. **Implementation climate:**The absorptive capacity for change, shared receptivity of involved individuals to an intervention, and the extent to which use of that intervention will be rewarded, supported, and expected within their organization. *Tension for change:*The degree to which stakeholders perceive the current situation as intolerable or needing change. *Compatibility:*The degree of tangible fit between meaning and values attached to the intervention by involved individuals, how those align with individuals' own norms, values, and perceived risks and needs, and how the intervention fits with existing workflows and systems. *Relative priority:*Individuals' shared perception of the importance of the implementation within the organization. *Organizational incentives and rewards:*Extrinsic incentives such as goal-sharing awards, performance reviews, promotions, and raises in salary, as well as less tangible incentives such as increased stature or respect. *Goals and feedback:* The degree to which goals are clearly communicated, acted upon, and fed back to staff and alignment of that feedback with goals. *Learning climate:* A climate in which: leaders express their own fallibility and need for team members' assistance and input; team members feel that they are essential, valued, and knowledgeable partners. **Readiness for implementation: **Tangible and immediate indicators of organizational commitment to its decision to implement an intervention. *Leadership engagement:*Commitment, involvement, and accountability of leaders and managers. *Available resources:* The level of resources dedicated for implementation and ongoing operations including money, training, education, physical space, and time. *Access to information and knowledge***: **Ease of access to digestible information and knowledge about the intervention and how to incorporate it into work tasks.
**Outer setting:** External influences on intervention which includes the features of the external context or environment that might influence implementation (patient needs and resources; cosmopolitanism degree to which an organization is networked with other external organizations); peer pressure (competitive pressure to implement an intervention); external policies and incentives (eg, policy and regulations, external mandates, recommendations and guidelines, pay-for-performance, collaboratives, and public or benchmark reporting).
**Process of implementation: **Includes strategies or tactics that have an influence on implementation, including planning, executing, reflecting and evaluating, and presence of key intervention stakeholders and influencers.

Abbreviation: CFIR, Consolidated Framework for Implementation Research. Source: Damschroder et al.^32^

###  Site Selection, Sampling and Recruitment

 The research was carried out in two Ethiopian cities in two different states, with the primary site being part of the first wave of implementation in regional cities and the second site part of the subsequent wave. The primary site was selected based on pre-existing relationships and interest by the Regional Health Bureau. The second city was selected based on feasibility and logistics factors; it was the closest city from another administrative state that was implementing the FHTs. In our study site, the only reliable FHT records available captured the results of the household survey, thus the poor level of FHT implementation was not known at the time of site selection.

 Administrative lists obtained from the Regional Health Bureau and snowball sampling were used to recruit administrators. In City 1, a total of four FHTs were trialled in one health centre covering three surrounding Kebeles. The four FHTs included 22 UHEPs and 6 facility-based health workers (FBHWs). We recruited all FHT members in City 1. In City 2, we interviewed a total of 9 FHT members and administrators who were present at health centres and offices during two days of visits. During that time, no competing or additional perspectives emerged that differed from City 1.

###  Data Collection

 The research team was not involved in the implementation of the FHT program. This study was carried out as part of a broader research programme examining innovations in urban CHW roles in LMICs.

 In order to gain collective insight about how the FHTs have affected the types of services provided and relationships with different stakeholders, we carried out two focus groups involving half of the UHEPs in the primary study site. Given the small number of program administrators (health centre, city administration, regional health bureau, Ministry of Health, NGO [non-governmental organization] partner) and FBHWs involved in the FHTs and the sensitive nature of sharing information about a federal initiative which was overall poorly implemented, we conducted semi-structured interviews with these groups. With those UHEPs not selected to the focus group discussions, we carried out individual interviews in order to inquire about personal experiences and perspectives regarding the FHT implementation that would not be well captured in a group setting. Interview guides inquired about: roles in implementing/overseeing the FHTs; preparatory activities, processes, and resources put in place to support implementation; program expectations, level of engagement and relationships between different cadres and stakeholders; and challenges encountered.

 We recruited local university staff as research assistants and provided a two-day orientation. Interview guides were translated into local languages (by Ethiopian co-author ME) and back-translated by research assistants. As is the practice in qualitative interview-based research, the interview topic guides were examined and adjusted in minor ways on an ongoing and iterative basis to: assess if the research questions were being understood and being appropriately addressed by respondents; to clarify emerging meanings; and to add/include additional topics/probes on important themes and topics that emerged during the interviews. Research assistants conducted interviews/focus groups in private offices or offsite locations to ensure privacy and independence. Based on local advice, participants were provided with honoraria (~USD $7 in mobile phone credit) to offset time and travel costs to interview locations. Written consent was obtained from participants. Conversations were audio-recorded and then simultaneously translated and transcribed by the research assistants. Debriefing sessions were held daily to discuss main findings and raise areas for new inquiry. Data collection was conducted from July-October 2019.

###  Analysis

 We used framework analysis to code data according to the CFIR domains.^33^ Findings were triangulated by analyzing responses within and between participant groups, and across the two cities. Of the five CFIR domains, participant responses pertained primarily to the inner and outer setting. We focus our presentation of findings on the domains most emphasized in our study context, as encouraged by the CFIR developers.^32^

 The primary author (TL) coded the data in NVivo 11 and conducted the primary analysis. Interpretation was validated by Ethiopian contributor (ME) who reviewed all transcripts.

## Results

 From the two cities, 18 program administrators (8 from health centres; 10 municipal/regional/federal/NGO representatives) were interviewed. Of the 34 participating FHT members, 9 FBHWs (5 from City 1) and 13 UHEPs (9 from City 1) participated in interviews. The remaining 12 UHEPs from City 1 were involved in 2 FGDs. There was one no-show among UHEPs and one FBHW was on long-term leave. Among participants, 1 administrator, 4 FBHWs, and all UHEPs were women. Most UHEPs were long-serving, with an average of 7 years of service.

 The following sections present findings on: (1) The extent of FHT adoption; and, (2) FHT member and administrator perspectives on factors affecting adoption. Responses are organized according to CFIR’s inner setting and outer setting domains.

###  I. Extent of FHT Adoption

 Given that the FHTs should officially have been operating in a routine manner, we sought participants’ perspectives on the extent to which they engaged in the FHTs. FHT members and (most) administrators reported that the FHTs were not functioning: “Now, we can say it is dead” (City1-FGD1). Respondents revealed that participation had been limited and short-lived in both study sites. According to respondents, FHTs had ceased operating within the first six months, with many FHT members indicating much earlier. Implementation from the onset was weak: teams were not well organized according to the expected number and composition of staff and few FHT household visits were conducted. For example, City 1 engaged only 6 FBHWs to participate in FHTs. Total number of reported FHT visits by UHEPs in the first 6 months ranged from zero to six and from two to ten for FBHWs. Respondents indicated that FBHWs had stopped participating, follow-up and support from the health centre had ceased, and FHT members had reverted to previous work patterns, including return of UHEPs’ duty station to Kebele offices. FHTs visits were by then only occasionally performed to correspond with external monitoring visits.

###  II. Inner Setting: Implementation Climate and Readiness

 After learning that the FHT approach had been abandoned as part of routine work, we examined what factors hindered its adoption. Factors which impacted ‘willingness to participate’ – the defining feature of the adoption phase – predominantly focused on inner setting CFIR domains, particularly in relation to ‘implementation climate’ and ‘implementation readiness.’ Analysis of participants’ responses suggest that implementation climate strongly affected FHT members’ participation while implementation readiness impacted administrator capacity and willingness to support the FHTs.

####  Implementation Climate 

 In the following sections, we show how features of the implementation climate differentially affected the two main FHT cadres, and by extension their willingness to engage. Divergent perspectives between FBHWs and UHEPs regarding FHT compatibility with existing roles, the need to modify current work modes, incentives, and the importance of outreach vis-à-vis clinic-based services shaped UHEP willingness and FBHW reluctance to participate.

####  i. Compatibility 

 Despite the fact that both FBHWs and UHEPs recognized the potential benefits of the FHT model for clients, UHEPs considered the FHTs to be highly compatible with their roles while FBHWs conveyed the opposite.

 FBHWs found the workflows associated with the FHTs were incompatible with clinical shifts and increased their workload:

*“The UHEPs usually think that we [FBHWs] don’t want to work outside like them. But we work in different shifts that are tiresome. For example, if I have a night shift and the FHT visit is scheduled the next morning, it’s impossible to do this work back-to-back” *(City1-FBHW5).

*“When the FBHWs went on FHT home visits, fewer providers remained at the health centre and they were not able to cover the high workload and patient flow. As a result, the FBHWs began complaining*” (Adminstrator-2).

 Responses from the FBHWs suggest poor compatibility is affected by external factors including intervention design and resourcing, and a culture of privileging clinic-based services over community outreach by health centre administrators and health workers whose roles have traditionally been based at health centres (discussed further in the subsequent section on ‘relative priority’).

 In contrast, the UHEPs anticipated a number of positive benefits to their workflow and scope of work. As illustrated in the following quotes, UHEPs expected that the FHT approach would support more manageable, structured, and effective work processes by: (1) limiting Kebele officials’ authority to assign non-health related tasks; (2) focusing specifically on high-need households rather than covering all catchment households; and, (3) offering treatment services to clients rather than health education and referral only:

*“They told us that our office will be at health centre and we expected that no Kebele leader will order us” (followed by laughter) *(City1-FGD1).

*“There are about 500 households in my catchment. In the past, UHEPs could not even get to all households within a year. But with the FHT, we prioritized which households to visit” *(City1-FGD1).

*“In this team, we treated those who were bedridden and affected by chronic diseases, sometimes providing 3 or 4 different services at a time which the patients found very helpful”*(City2-UHEP1).

 Even after the FHTs dissolved, UHEPs still spoke favorably of the work benefits that they had briefly experienced. However, as the FBHWs, who served as technical leads, were unwilling to participate, the FHT visits could not continue. In an effort to maintain some of the FHT-related work benefits, some UHEPs continued carrying out their work in groups even without FBHW participation.

####  ii. Impetus for Change

 While the FHTs were specifically designed to redress challenges in the UHEP work environment, the impetus for change was one-sided without corresponding strategic benefits to FBHWs’ service delivery roles. Although drawing FBHWs into the community was an objective of the FHT reform, no FBHWs identified lack of community engagement as problematic. At best, working in the community was perceived as peripheral benefit: “I don’t receive any special benefit, only the satisfaction of serving my people” (City2-FBHW1).

 In contrast, UHEPs clearly articulated how they expected FHTs to mediate challenges around poor receptivity, lack of professional validation, and professional stagnation, among others:

*“The advantage of the FHT is that it will help improve our acceptance. The community will start seeing us as health professionals, not merely as Kebele workers” (City1-UHEP9).*

*“Although I am a clinical nurse by profession, our job mainly focuses on disseminating health information. As a result we feel far from our profession, but the FHTs will give us the opportunity to practice clinical work” *(City1-UHEP6).

*“Most of us have been working in the Kebele for more than five years and the usual health extension work has grown tiresome” *(City1-UHEP8).

 While FBHWs recognized the advantages to UHEPs, they did not see the FHTs as a strategic intervention to improve their own working conditions.

####  iii. Organizational Incentives and Rewards

 As shown in the participant responses below, UHEPs appeared motivated by non-financial, work-related benefits presented by the FHTs, while lack of monetary incentives was a major inhibitor of FBHW participation in the absence of other benefits.

 FBHWs believed they should be compensated for the additional burden that fell outside their normal work scope and schedule:

*“It was tiresome to go to the community without transportation as we were used to working only at the facility. I had to work extra hours to conduct home visits in the morning and then complete my normal health centre shift in the afternoon. I had to do both shifts without additional payment. As a result, we [FBHWs] complained a lot”* (City1-FBHW1).

 In contrast, UHEPs felt ‘happy’ and ‘lucky’ to participate in the FHTs, despite the lack of financial incentives (City1-FGD1). Even within the first few visits, UHEPs experienced positive outcomes of the FHTs, particularly around improved community receptivity: “Before the FHTs, communities did not accept us because they were tired of hearing only about prevention. But when they started to get medical services, they began to respect us” (City2-UHEP2). These impressions were echoed by FBHWs. UHEPs also experienced early indications of improved community-to-facility referrals: “We had been doing referrals before the FHTs, but with the FHTs, we achieved better results. For example, cervical cancer screening performance reached its peak when we started FHTs” (City1-FGD1).

 While UHEPs had repeatedly asked their supervisors to re-activate the FHTs, FBHWs across the board reported that they were not interested in regularly participating in the FHT as currently structured. FBHWs did not perceive any need to change their service delivery models and considered the FHTs an extra work burden without additional payment. After a few FHT visits, the FBHWs stopped participating.

####  iv. Relative Priority

 In the context of the issues raised above, FBHWs continued to give priority to clinic-based work over outreach. Despite the fact that FBHWs understood the benefits of the FHTs for clients and in some cases acknowledged their own responsibility to support outreach services, in practice the dominant culture of prioritizing clinic-based services continued to drive activities:

 “*We came to understand that the FHT program is very important for making health services accessible to families. The FHTs help minimize travel time to health facilities, transportation costs and other treatment costs by providing disadvantaged individuals with all the services in their home*” (FBHW-2). Another FBHW illustrated how being involved with the FHTs help shift their mindset: “*Previously we used to believe that the outreach program was the responsibility of the health extension workers and their supervisors. After the FHT program was implemented, we realized that we have to support the community…it helped us be empathetic to those who were in need*” (FBHW-1). However, in practice, continued prioritization of clinic-based work appeared to be reinforced by prevailing work hierarchies and culture in which outreach services were perceived as supplementary rather than a core responsibility:

 “*We are expected to engage in the formal jobs that we are employed for” *(City1-FBHW3).

*“When UHEPs complain that we do not accompany them, we tell them that the patients can always find us at the health centre whenever they want; but if we are conducting FHT visits, the other patients can’t access us” *(City1-FBHW5).

 Consequently, FBHWs reported never or rarely attending weekly FHT review meetings. Health centre administrators similarly gave higher priority to clinic-based services: “*there is high workload among the health centre staff…we have to first provide health services to those who come to the facility rather than focusing on those who prefer to remain home*” (Administrator-2). Accordingly, administrators reported re-assigning FHT designated workspaces for other purposes. Without office space, UHEPs were forced to return to Kebele offices. UHEPs lamented that “*after moving back to Kebele offices, the linkage with the health centre became minima*l” (City1-UHEP1). Kebele administrators also prioritized other UHEP work over FHT visits. A FBHW who previously had a role in UHEP supervision noted that “*The FHT was controlled and monitored by [NGO], while the UHEPs’ work was monitored by the town’s health department. So, more focus was given to other UHEP roles*” (City1-FBHW3).

 In sum, no actors except the UHEPs – the least powerful group – prioritized the FHTs.

####  Implementation Readiness 

 While poor implementation climate was an important determinant of non-adoption, respondents also pointed to broader constraints around capacity, resourcing, and leadership. The following section examines issues related to implementation readiness from the perspective of administrators, while also outlining the impacts poor readiness had on FHT members.

####  i. Available Resources

 The FHTs were resource-intensive and neither city was able (or willing) to provide the necessary resources. Administrators acknowledged “*the program stopped in the first half of the year because it requires huge support*” (Administrator-14). Staffing limitations and lack of medicines and diagnostics were identified as the primary constraints, with lack of office space and transportation also hindering implementation.

 Administrators raised feasibility issues around the human resource requirements: “*Now we are implementing in only 3 Kebeles. I think it will not be possible to implement in all 12 Kebeles as we will end up with no health workers in the health centre*” (Administrator-11). As a result, FBHWs were only assigned to FHTs on an ad hoc basis.

 Lack of dedicated budget and a defined logistics chain for community-level supplies were identified as challenges. Inability to consistently provide free medicine and assistive equipment to vulnerable households contributed to feelings of uselessness among FHT members: “*FHTs require extra budget. Some households may require other supports like wheelchairs. The households keep asking us for ‘real’ help rather than only assessing their problems*” (City2-FBHW1). Some FHT members reported buying medicines from their own pockets for patients who are “*powerless to buy drugs and are crying out for help*” (City2-FBHW1). Although the NGO partner had initially supplied some diagnostic equipment (eg, blood pressure monitors; glucometers; first aid kits), availability and functioning of the equipment became problematic: “*We use the glucometer from the health centre. So when we take the glucometer to the community and patients in the health centre require it, it becomes a challenge*” (City1-FBHW5).

 Lack of rooms at the health centre to serve as UHEP offices, carry-out FHT review meetings, and provide a dedicated outpatient room for FHT-referred clients also impeded FHT functioning. Administrators indicated these infrastructure requirements were beyond their budget and accommodating so many UHEPs within one health centre was not feasible.

 Lack of transport was a source of complaints by FHT members, and negatively affected FHT efficiency and patient referrals. Citing budget constraints, administrators had not provided the prescribed transport support: “*In the original implementation in Addis Ababa, there was support for transportation and airtime [credit for mobile phones] to communicate during the referral process. But we did not provide this type of support and feared the FHT members might get offended if they found out*” (Administrator-4).

 Insufficient resourcing impacted capacity and willingness to participate both from the FHT members and the responsible administrators.

####  ii. Leadership Engagement

 Respondents from all groups of participants pointed to a lack of political commitment across all levels of local administration for the FHT program, contributing to poor ownership, internal resource mobilization, and coordination. A common sentiment was that “*no one is taking responsibility for the program*” (City1-FBHW1). Respondents pointed fingers at different administrative levels. While some blamed resourcing issues on the city health department “*The health centre can’t solve the challenges related with infrastructure and room shortages. The city health department needs to solve it*” (Administrator-2), others highlighted the lack of internal resource mobilization by health centres: “*Despite the shortage of rooms in health centres, they should attempt to efficiently share them. They should also invest their internal financial resources from service charges and consider this program like other activities managed by the centre*” (Administrator-10). Other administrators acknowledged that the FHTs were more successful in another city where there was strong support from the town office, including a dedicated budget and additional hiring to support the FHTs (Administrator-13).

 Respondents outlined how low commitment and contested governance of the UHEPs contributed to poor coordination of the FHT program. Respondents stated that “*They are giving little attention to FHT and there is poor follow-up. The command chain from the regional health bureau to city health department to health centre and to Kebele-level is very weak*” (Administrator-3). Respondents noted that loss of control over UHEPs was contested by Kebele officials, with weak transfer of responsibility to health centre administrators, resulting in poor oversight: “*Previously, the UHEPs were based at and reported to the Kebele administration. As a result, the health centres were not providing support and following UHEP activities properly. They only come together during review meetings*” (Administrator-4). Respondents indicated that even these joint meetings soon faded out.

 Other root causes of poor leadership were attributed to the top-down, federally-imposed mandate discussed in the next section.

###  III. Outer Setting: External Origin of Intervention and External Policies 

 As adoption occurs at the organizational level, we first presented determinants of FHT adoption related to the inner setting. However, as features of the implementation environment are also shaped by external factors, we now examine how national policy and country context influenced FHT adoption.

 The FHTs were perceived as federally imposed, lacked clarity on resourcing and governance, and failed to politically or financially motivate local leadership. As a result, administrators noted that the FHTs did not get much buy in at lower levels, despite high levels of enthusiasm at the federal level (Administrator-12). Administrators described the federal government and NGO partner as the ‘primary implementers’ and indicated that they were only implementing at the federal government’s direction (Administrators-12/13). FHT members noted that FHT visits were primarily organized for external officials and that “*there was no one to take responsibility for handling the FHT implementation. Only [NGO] was working with us*” (City1-UHEP8/FBHW1).

 Weaknesses in the federal FHT policy, particularly clarity around resourcing, contributed to implementation challenges. When supply kits fell into disrepair, a Kebele official stated that “*There is no clearly stated responsibility on who should avail these materials*” (Administrator-6). Respondents also indicated that the policy was unclear about: (1) What revenue streams should be used to finance community provision of medicines; (2) Whether national standards for staffing ratios were sufficient to meet FHT requirements; (3) How to integrate multisectoral collaboration given the absence of social workers and budget for social supports; and, (4) The expected balance between FHT activities and other activities previously conducted by UHEPs.

###  IV. Other Factors

 Compared to the inner and outer setting, respondents commented little on other implementation domains (intervention characteristics, characteristics of individuals, implementation process). Other factors reported to a lesser extent included: training issues (too short; not delivered in a timely manner); lack of clarity about FHT member responsibilities (individual roles during interactions with clients; frequency of visits); weak pre-existing connections between UHEPs and FBHWs (UHEPs in the capital city were based in health centres prior to the FHT pilot, whereas in regional cities UHEPs are typically based in the Kebele); and poor time management (households not alerted in advance; wasted time gathering team members). While political instability in the region was not explicitly mentioned, we acknowledge that it likely contributed to low engagement by local leadership.

###  Summative Findings

 Implementation experiences and challenges were similar in both study sites across two administrative regions. The absence of financial incentives, combined with clinical shift incompatibility and no perceived need to change their service delivery roles, reinforced FBHWs’ unwillingness to participate. At the management level, lack of leadership contributed to limited resource mobilization, exacerbating the challenges of a resource-intensive team model. Lack of drugs and challenges in fully staffing the teams contributed to a sense of ineffectiveness, further decreasing the willingness of FBHWs and administrators. Table 2 demonstrates the skewed distribution of perceived benefits and costs across groups of participants. Following the phase-out of NGO-led technical assistance, the FHTs quickly became non-operational. While the following results focus on our two study sites, anecdotal evidence from administrators with cross-city program oversight indicated that the implementation was challenging across most sites, though one city not examined in this paper appeared to interviewees as more committed to resourcing and delivering the FHTs.

**Table 2:** Perceived Benefits and Costs of FHT Implementation (According to Participants)

	**Perceived Benefits (Intangible)**	**Incentives (as Reported)**	**Perceived Costs (Financial, Workforce, Opportunity Costs)**
UHEPs	High potential benefit: Enhanced scope of work Greater client satisfaction More manageable workload Professional validation Professional development	No regular incentives provided	Low cost: Minimal interference with current work
FBHWs	Low benefit: Intrinsic reward of providing societal service	No regular incentives provided	High cost: Increased work burden Reliance on private transport
Kebele officials	No benefits identified	No incentives reported	High cost: Loss of control over UHEP tasks
Health centre administrators	No benefits identified	No additional resources for hiring extra staff	High cost: High resource needs: rooms, staffing, budget for transportation, medicine, and supplies

Abbreviations: FHT, family health team; UHEPs, urban health extension professionals; FBHWs, facility-based health workers.

## Discussion

 With increasing endorsement of CHW-integrated healthcare teams,^10,11^ such teams may represent an attractive model for LMICs seeking to simultaneously tackle significant health disparities in vulnerable populations and provide professional development opportunities for large, established CHW cadres. However, healthcare teams are difficult to implement.^14-16^ With limited literature on CHW-integrated healthcare teams, lessons from our study of poorly functioning FHTs in Ethiopia raise important policy considerations for LMICs regarding implementation climate and readiness. While healthcare team studies from high-income countries have predominantly focused on interpersonal dynamics^8,34^ our study’s unique focus on teams which operated only briefly before fading out, highlight important organizational and system determinants that influence the critical stage of adoption. In using the CFIR to examine common determinants across our two study sites and the interactions between determinants in the inner and outer setting, we found that: lack of incentives at multiple levels (financial, political, and professional) strongly affected willingness of clinicians and administrators to participate in FHTs, while resource constraints significantly affected capacity to implement. Other studies from Africa have similarly found that implementation of CHW-integrated teams is affected by resource constraints and by tensions related to changes in the autonomy and power of different players.^35-37^ Our study adds to the literature by helping to conceptualize the linkages between these implementation factors. In considering whether to initiate CHW-integrated team models, policy-makers need to carefully assess resource requirements, and put in place mechanisms to motivate engagement by team members and their supervisors. Otherwise, the creation of teams is unlikely to be productive for health workers or for clients, as shown in our study. Below we discuss how willingness and capacity interacted with each other and with an LMIC context to negatively impact adoption.

 Implementing healthcare team models represents a substantial organizational change process,^38^ and is typically accompanied by unequally distributed gains and losses for different parties which can contribute to uneven buy-in,^17^ as seen in our study. While Ethiopia’s team model successfully tapped into UHEP professional and performance motivations, the absence of well-developed change management strategies aimed at clinicians and administrators led to poor overall adoption. For UHEPs, the FHT design was highly compatible with existing roles and drew on mechanisms known to motivate CHW engagement: anticipation of improved sense of legitimacy, value by community, and relatedness with health facilities.^39^ However, the FHT design and roll-out lacked intentional strategies for tackling elements of clinical work practice known to produce resistance (eg, differences between cadres related to hierarchy, power, and professional value systems, including pervasive culture of medical dominance^19,40^) and lack of carrots and sticks (eg, incentives,^14^ appraisal systems^17^) that are important for engendering participation. Clinicians felt the FHTs offered no professional benefit to them and did not see much reason to participate. As shown from the organizational development literature, without a ‘felt need’ for change, instituting change processes can be difficult.^18,21^ Reviews of facilitators and barriers in healthcare teams show that while health professionals often understand the value of collaboration,^17^ they can be reluctant to engage when new ways of working clash with their existing professional experience, including in our setting where clinicians believed they should focus on serving clients in a clinical setting.

 To support the adoption of healthcare teams, intentional change management strategies at the individual and organizational level are needed to equip and motivate staff to adapt ways of working. It is well-established that “professionals will not collaborate if the effort is only based on the notion that it will be good for clients.”^41^ Common experiences of healthcare teams across low- and high-income countries shows that orienting policies, communications, and incentives to support and reward collaborative practice in clinical settings is essential.^42^ Before initiating healthcare teams, policy-makers and program implementers need to establish: clear policies and protocols on team member responsibilities; orientation and ongoing training for teamworking; regular patterns of communication and supervision that are institutionalized; and formal evaluation.^17,42^ These enabling features were not observed in the FHTs, likely contributing to their poor adoption. While FHT clinicians vocally called for financial incentives, it will be important for LMICs to look at a range of strategic actions that tap into professional motivations (such as those mentioned above), given financial constraints and evidence that monetary incentives alone does not influence health worker performance.^43,44^ While reviews have shown that some teams are able to achieve successful, non-hierarchical ‘interdependence’ of CHWs and clinicians, cross-analysis of these positive case studies is needed to understand the enabling conditions for their success and provide lessons for initiating teams.^8^

 Establishing accountability mechanisms is essential for ensuring that team collaboration takes place.^14^ While the FHTs’ new governance structure recommended co-location of team members – a factor known to support team models^17,45^ – the new governance arrangements held little financial or political appeal for administrators. The new arrangements divested Kebele officials of responsibility for a core cadre used to carry out local political and development priorities. At the same time, the FHTs shifted responsibility of an entire cadre of workers to health centre administrators who had no clear interest in managing them, particularly without additional infrastructure and budgetary support. On the one hand, our findings show that when traditional CHW accountability and reporting systems are led by those external to clinical operations, establishing leadership and accountability for teamwork between clinicians and CHWs may be particularly challenging. Such challenges have also been observed in other African contexts when NGOs and district officials, who had previously held considerable autonomy over CHW activities, were requested to support team approaches.^35,36,46^ On the other hand, experience from the United States suggests that maintaining a supervisor outside of the clinic who understands community work has been a design strength.^47^ While targeting administrative culture is contested and usually bypassed by performance interventions in LMICs, there is a need to understand how strategic governance arrangements and distribution of responsibilities across actors can be leveraged to generate intrinsic motivation and reduce reliance on financial incentives.^48^ Research by the World Bank on publicly-administered community health interventions demonstrates that strategic communication tailored to local political institutions and tactical choices about resource control between different parties can shift power dynamics, tap into political motivations, drive internal resource contributions, and turn poor performers into high achievers.^48,49^ Such lessons and approaches need to be brought to bear in LMIC healthcare team models. Experience from South Africa shows that when districts and health centres had more ownership and flexibility in the design and implementation process, there was less disruption to existing organizational structures and less resistance to the reforms needed to support teamworking^36^; health district managers in charge of implementation advocated for a decentralized approach.^46^

 Healthcare team approaches are significantly affected by the level of resources dedicated to support them.^14,17^ Resource levels determine team composition, physical spaces available for teamworking, and other support available to teams.^17^ All of these factors severely hindered FHT implementation, with staff shortages the most emphasized. FHTs suffered from: insufficient numbers of clinicians generally and of particular cadres (doctors, pharmacists, social workers) to assign to outreach teams; lack of financing for providing medicine free of charge during FHT visits; insufficient stock of working diagnostic equipment to use in the community; no transport vehicles; and, lack of space to co-locate UHEPs at health centres and provide team meeting rooms. Together these factors rendered the teams largely ineffective and further decreased commitment. Similar constraints related to leadership, staffing, infrastructure, and equipment have been found to contribute to implementation challenges and decreased effectiveness of CHW-integrated healthcare teams elsewhere in Africa.^35,37,50^ Team working often requires additional inputs to offset increased work burden, which can otherwise strain existing resources and contribute to decreased motivation.^50-52^ In initiating community-based team models, LMICs should establish local staffing thresholds (minimum staff numbers and ratios between cadres) required to initiate teams and establish financing streams at the national, municipal, and health facility-level to meet resourcing requirements imposed by the team model. Our study highlights that lack of clarity about how health facilities are to meet the extra staffing and other resource requirements imposed by the FHT contributed to low buy-in by local administrators.

 In Ethiopia, we found that capacity barriers were strongly linked with health system constraints common to LMICs: financing gaps, low numbers of physicians overall and relative to large CHW cadres, weak supply and logistics systems (drug supply, diagnostics, transportation), and reliance on external implementing partners. Thus, factors in the broader implementation environment affected FHT adoption. Budget and workforce size are important determinants of team model outcomes in healthcare.^53^ Despite evidence that healthcare teams can improve management of chronic diseases,^54^ the fact that only about 6% of low-income countries have essential equipment necessary to carryout standard chronic disease tests and measurements at the primary care level raises serious capacity concerns for LMICs.^55^ Middle-income countries which have managed to establish team-based practices system-wide, such as China, Brazil, and Thailand, have invested significantly in basic primary health centre capacity and health workforce expansion.^56-58^ Without strategic investments in primary health systems to support readiness, simply reorganizing health workers into teams is unlikely to be effective or sustainable.

 Collaborative processes in healthcare are initiated for two purposes: to serve client needs and to serve professional needs.^41^ Ethiopia’s FHTs were initiated, in part, to redress UHEP work conditions that contributed to poor performance and motivation – issues common to many CHW programs globally.^59^ As the nature of geographic and social community changes with urbanization and economic development in LMICs, current CHW roles may not be productive or rewarding.^60^ Healthcare team models present one configuration for re-engineering how CHWs interface with clients and health systems, and by extension, shift CHWs’ experiences in performing their roles. In changing workplace relationships and conditions, team models have been shown to improve job satisfaction and retention among healthcare workers.^13,42^ Further, linking CHWs to formal health services is known to empower and motivate CHWs.^61^ Given the staffing and other resource shortages faced by LMICs, it may be more practical for LMICs to first initiate healthcare teams in urban settings which offer larger numbers of healthcare workers and better-equipped facilities in closer proximity. In South Africa, travel to remote areas hindered the frequency and efficiency of team-based services.^37^ However, it is clear that beyond staffing and facility resources, intentional change management strategies must be instituted to support team creation, without which, the benefits of healthcare teams will not materialize.

###  Limitations

 Our study is among the first to examine organizational and system-level factors that affect implementation of CHW-integrated healthcare team models in LMICs. Using a comprehensive framework, we compared experiences from two cities in separate administrative regions which revealed similar constraints. As key challenges were strongly associated with health system constraints (resourcing, workforce size, NGO-driven), we believe these factors are likely to be relevant for LMICs generally. Our study could be strengthened by further investigation of outer setting factors which did not feature prominently.

 Given the weak implementation in both sites, we could not examine: enabling conditions for adoption, community perspectives, and team dynamics. Future LMIC-based studies should investigate: strategies for promoting health system readiness for team models; effective change management strategies; and health worker/client outcomes.

## Conclusion

 Rethinking primary care service delivery models in LMICs is needed. Urban health needs are changing, and so are the expectations of the population and health workforce. Team-based healthcare models, including those involving CHWs, may be part of the solution, but careful consideration of health system readiness and effective organizational change strategies are needed to support their implementability and feasibility in LMICs. Exploring how to capitalize on large CHW workforces will be an important consideration for LMIC team models in healthcare. If Ethiopia is at all indicative of the LMIC experience, CHWs are likely to jump at the opportunity to be part of healthcare teams. The lynchpin will be getting all the other actors on board.

## Acknowledgements

 We would like to express our gratitude to the people who went above and beyond during the fieldwork in Ethiopia, particularly Mahteme Feleke Debela, Fanuel Belayneh, and Tesfaye Tufa for your research assistance, Fikadu Reta, Melisew Dejene, and Logan Cochrane for your support and troubleshooting, and Anteneh Asefa Mekonnen who made it all possible.

## Ethical issues

 Ethical approval was obtained from the SNNPR State Health Bureau’s Health Research Ethical Clearance (reference: £’-6-19/37453) in Ethiopia and from the University of Melbourne (reference: 1954330). Written consent was obtained from all participants.

## Competing interests

 The authors have no competing interests to declare. ME is an employee of the SNNPR State Health Bureau, Research and Technology Transfer Office in Ethiopia. His role is to contribute to independent assessment of health programs in the region. He did not have a role in FHT implementation and does not have any professional relationships with those involved in FHTs. He did not conduct any interviews or focus groups, but helped ensure good cultural translation of the tools into local languages, reviewed anonymized transcripts, and validated interpretation of findings.

## Authors’ contributions

 TL led the study design, data collection, analysis and write-up. ME, BM, AM, SK, and MK contributed to the study design, interpretation of results, and manuscript revisions. All authors read and approved the final manuscript.

## Funding

 This work was supported by a Melbourne Research Scholarship (University of Melbourne) and the Canadian Institutes of Health Research (Scholarship Award 164268). Funding bodies did not have any role in the study design or the collection, analysis, and interpretation of data.
